# Editorial: Inflammatory and inflammatory-like responses in insects

**DOI:** 10.3389/fimmu.2023.1184429

**Published:** 2023-04-18

**Authors:** Akira Goto, Shanming Ji, Stanislava Chtarbanova, Takayuki Kuraishi

**Affiliations:** ^1^ Institut National de la Santé et de la Recherche Médicale (INSERM), Paris, France; ^2^ Insect Models of Innate Immunity (M3I; UPR9022), CNRS, Strasbourg, France; ^3^ Université de Strasbourg, Strasbourg, France; ^4^ Anhui Agricultural University, Hefei, China; ^5^ University of Alabama, Tuscaloosa, AL, United States; ^6^ Kanazawa University, Kanazawa, Japan

**Keywords:** drosophila, inflammatory response, insect, metabolism, stress response, omics, humoral (innate) immunity, cellular innate immune response

Inflammation is the host reaction against various external and internal stimuli such as pathogen infection, tissue injury and/or dysregulated immune response. Numerous studies mostly from mammals have identified several crucial sensors and associated downstream signaling pathways that evoke inflammatory responses. Nonetheless, a full understanding of the underlying molecular mechanisms is still far from understood, because of the complex cross-talk between the innate/adaptive/stress immune responses and the implication of other biological processes such as metabolism. Insects solely rely on innate immunity and have contributed numerous important findings in the field, having a high potential to uncover new aspects of inflammatory reactions.

This Research Topic explores recent advances regarding inflammatory-like reactions in insects. As Research Topic Editors, it was our great pleasure to curate and review a number of interesting manuscripts in which cover a wide range of innate immune and inflammatory-like responses across different infection models and versatile methodologies. In this Editorial, we would like to summarize the main findings and perspectives from the accepted articles in chronological order.

Caravello et al. systematically investigated the contribution of a wide range of *Drosophila* immune defenses following infection with the obligate intracellular parasite *Tubulinosema ratisbonensis* (*Microsporidia* group). Results from the functional genetic approach indicated that phagocytosis acts as a resistant defense mechanism to control the parasite infection. In contrast, surprisingly, many of the classical antimicrobial defenses such as the systemic immune response mediated by the Toll and IMD pathways, extracellular reactive oxygen species, thioester proteins, xenophagy and intracellular antiviral response pathways were not involved in the resistance. Several genes such as *PGRP-LE* and *Prophenoloxidase1* (*PPO1*) were found to promote parasite replication, while and *PPO2* and *Eiger* were involved in resilience/disease tolerance. In conclusion, this study provided a large unexpected view of classical innate immune defenses to infection with *T. ratisbonensis*.

Using *in vivo* Zika virus (ZIKV) infection model in *Drosophila*, Tafesh-Edwards et al. examined the host metabolic changes to the ZIKV infection. They found that flies deficient for *Dicer-2*, a central component of the antiviral RNAi pathway exhibit dysregulated glucose and glycogen metabolism and reduced insulin signaling pathway. Accordingly, insulin receptor substrate *chico* mutants displayed higher susceptibility and elevated ZIKV copy numbers, suggesting Dicer-2 is involved in insulin-mediated antiviral response to the ZIKV infection. Interestingly, the data also showed that these phenotypes are more severe in females than males, proposing that sex-dependent metabolic changes should be considered in the rates of infection and susceptibility to the progression of ZIKV-mediated disease.

Indeed, it is still poorly characterized how animals adjust their lipid metabolism in response to pathogenic infections. Deng et al. performed an integrated time-course transcriptomics and lipidomics analysis and elegantly demonstrated that ergosterol, the main sterol in the plasma membrane in flies, promotes host defense against infection with the mild-pathogen Gram-negative bacterium *P. carotovorum* (synonym: *Erwinia carotovora*, *Ecc15*). In particular, artificial increase of ergosterol by oral feeding or genetic depletion of Acsl, a long-chain fatty acyl-CoA synthase, significantly enhanced host survival rate to bacterial infection. Thus, this study revealed a critical role of lipid metabolism adaptation to bacterial infection and shed a light on a previously unidentified Acsl-ergosterol metabolism in innate immune response.

Hua et al. provided a more mechanistic insight how the IMD pathway is negatively regulated by the deubiquitinase (Dub) dTrbd which targets K63-linked ubiquitination of dTak1. Using Co-IP and GST pull-down assays, the authors showed that the N-terminal NZF domain of dTrbd is required for a physical interaction with dTak1. Ubiquitination/deubiquitination assays and phase separation analyses demonstrated that the Linker region (LR) of dTrbd modulates its condensation and contributes to regulate the Dub enzymatic activity. Interestingly, authors also observed that dTrbd/dTak1 interaction and dTrbd condensation and its Dub enzymatic activity are enhanced upon bacterial infection, suggesting dual mechanisms of dTrbd to regulate the IMD pathway activation.

Finally, Vaibhvi et al. ambitiously tested the hypothesis that two innate immune cell types, namely the fat body and hemocytes, show similar immune responses to a systemic bacterial infection. In spite of a technical difficulty to isolate adult hemocytes, authors succeeded in setting up the experimental conditions and performed transcriptomic analysis on both cell types. This comprehensive analysis surprisingly found that only 10% of genes is shared between hemocytes and fat body cells, and that genes are mainly categorized into classical immune effectors or antimicrobial peptide (AMP) genes. Fat body-specific differentially regulated genes were found to involve in translation and protein export along with GO terms of oxidative reduction and phosphorylation, which is indicative of energy-consuming and metabolically active status of the fat body. In contrast, hemocytes showed a signature related to phagocytosis. In conclusion, two major immune competent cell types showed a different response to infection, aligning well with the specific tasks of humoral and cellular immunity, respectively.

The findings of these studies significantly change the concept of classical innate immune responses and extend to a better understanding of insect inflammatory-like reactions. We appreciate all the excellent contributions to our Research Topic, which we hope will stimulate novel and exciting studies in the field of insect immunology.

## Author contributions

AG wrote the editorial with SJ, SC and TK. All authors approved the submitted version.

